# 772. Learning from our patients: An exploratory study to inform the development of a case tracking dashboard for subspecialty fellows

**DOI:** 10.1093/ofid/ofad500.833

**Published:** 2023-11-27

**Authors:** Daniel J Minter, Annabel K Frank, Brian S Schwartz, Sirisha Narayana

**Affiliations:** UCSF, San Francisco, California; UCSF, San Francisco, California; University of California, San Francisco, San Francisco, CA; UCSF, San Francisco, California

## Abstract

**Background:**

Educators identify case tracking (following-up prior patient encounters) as a valued practice to cultivate physician expertise. Much of the existing research has focused on the practices of general medicine providers, where challenges include insufficient time and difficulty tracking patients in the electronic health record (EHR). Subspecialty fellows constitute a group of advanced learners who see a high volume of patients and need to quickly become subject matter experts, yet little is known about their particular needs and motivations around case tracking. In this study, we performed a needs assessment with fellows to inform the design of an EHR-based case tracking dashboard that can facilitate learning from prior patient encounters.

**Methods:**

We conducted semi-structured interviews with 22 medicine subspecialty fellows (including 4 Infectious Diseases [ID] fellows) at a single academic medical center that explored participants’ motivations, preferences, and practices around case tracking. Interview transcripts were coded and analyzed with directed qualitative content analysis. Results of this analysis will be used to inform the design of a subspecialist EHR-based case tracking dashboard.

**Results:**

Participants were motivated to engage in case tracking, with curiosity regarding patient outcomes as a strong motivator. Although some attempted to track all consults, most focused on subsets of patients, feeling that not all encounters were of equal educational value. Participants highlighted particular results, specialty-specific notes, and major outcomes as the most useful information. ID fellows specifically were interested in post-discharge ID clinic encounters, send-out tests, and outcomes of their management decisions. Despite widespread motivation to engage in case tracking, manually adding patients to lists and remembering to follow up poses logistical and time barriers.

**Conclusion:**

An ideal EHR case-tracking dashboard would automatically keep a list of patients cared for by fellows and provide easy access to own-specialty notes, specific results of interest, and information on the global clinical course. The ability to highlight specific cases anticipated to have more educational value would also be of benefit.

**Disclosures:**

**All Authors**: No reported disclosures

